# Comparative Analysis of Intestinal Microbiota Between Tetrodotoxin-Containing and Tetrodotoxin-Free *Takifugu rubripes*

**DOI:** 10.3390/md23040140

**Published:** 2025-03-24

**Authors:** Hanyuan Zhang, Jilun Hou, Youxiu Zhu, Biyin Wu, Jiangong Ren, Zhaohui Sun, Xia Liu

**Affiliations:** 1 Key Laboratory of Aquatic Genomics, Ministry of Agriculture and Rural Affairs, Beijing Key Laboratory of Fishery Biotechnology, Chinese Academy of Fishery Sciences, Beijing 100141, China; 2 Beidaihe Central Experiment Station, Chinese Academy of Fishery Sciences, Qinhuangdao 066100, China

**Keywords:** *Takifugu rubripes*, tetrodotoxin, 16S rRNA sequencing, intestinal microbiota, microbial function

## Abstract

Tetrodotoxin (TTX) is a potent marine neurotoxin found in pufferfish, causing severe poisoning or death if consumed improperly. Studies have indicated that intestinal symbiotic microbiota are associated with the production and accumulation of TTX in pufferfish. However, the specific symbiotic microorganisms involved in these processes and their respective functions remain unclear. This study explored differences in intestinal microbiota related to the TTX content between toxic and non-toxic tiger puffer *Takifugu rubripes*. We found that the dominant phyla exhibiting significant abundance differences between the two groups were Proteobacteria and Bacteroidota, with the core bacterial genera being *Rikenella*, *Vibrio*, *Photobacterium*, and *Bacteroides*. Moreover, the genera *Marinimicrobium*, *Idomarina*, *Galbibacter*, and *Brumimicrobium* were reported for the first time to be potentially associated with TTX bioaccumulation in *T. rubripes*. In addition, an integrated analysis with our previous study indicated that the “ABC transporters” pathway may play significant roles in the production and transport of TTX in both symbiotic microorganisms and *T. rubripes*. This study preliminarily investigated the intestinal symbiotic bacteria associated with the accumulation and metabolism of TTX in *T. rubripes*, as well as screening potential microbial biomarkers for assessing the safety of pufferfish.

## 1. Introduction

Pufferfish is a widely distributed fish species worldwide, existing in both seawater and freshwater. Due to its tasty flavor and high nutrition, pufferfish is a well-received high-end food ingredient and an economically important breeding species in East Asian countries [1]. However, the presence of tetrodotoxin (TTX) in pufferfish has been a significant constraint on the further advancement of the pufferfish aquaculture industry. For a long time, incidents of food poisoning caused by improper consumption of pufferfish have occurred and been reported, with severe cases leading to death [2,3].

TTX is a highly potent marine bio-neurotoxin [4], which is a kind of alkaloid with a small molecular weight (chemical formula: C_11_H_17_N_3_O_8_, molecular weight: 319.27) and high biological activities [5]. It is a typical voltage-gated sodium ion channel (Na_V_) blocker since it selectively binds to Na_V_ and exerts toxic effects by inhibiting nerve and muscle conduction [6]. TTX has been used in medical research for the treatment of various diseases such as analgesia, anesthesia, anti-cancer, cardiovascular disease, and neurological disorders according to its mechanism of action [7,8,9,10,11,12]. There are a variety of naturally occurring TTX analogues [13]. So far, more than 20 TTX structural analogues have been reported with different levels of toxicity [14,15,16,17]. The significant differences in the toxicity among TTX and its analogues were found to be positively correlated with the number of hydroxyl groups [18]. TTX was firstly isolated and reported in Tetraodontidae [19], and subsequent studies have found that TTX widely exists in organisms with different evolutionary levels, including algae, parasites, mollusks, crustaceans, echinoderms, amphibians, etc. [20].

The TTX content of pufferfish varies remarkably under different habitat conditions; however, the origin and accumulation pathways were not clear yet. There are currently three main hypotheses regarding the origin of TTX in pufferfish: the exogenous hypothesis (via food chain enrichment), the endogenous hypothesis (self-transformation and accumulation of TTX by pufferfish), and the endosymbiotic origin hypothesis (the production of symbiotic microorganisms) [21]. Three hypotheses attempted to explain the possible origin of TTX in pufferfish from different perspectives [22]. Previous studies have demonstrated that tiger puffer (*Takifugu rubripes*) have the ability to convert TTX analogues or precursors enriched by the food chain or produced by symbiotic microorganisms into TTX, then transport and accumulate it to different tissues and organs [23,24]. Since the endosymbiotic origin hypothesis was proposed, researchers have successively isolated bacteria and actinomycetes that could ferment to produce TTX analogues from various TTX-containing animals [25]. Thus far, the bacterial species that can produce TTX and its analogues mainly are *Vibrio*, *Pseudomonas*, *Photobacterium*, *Aeromonas*, *Plesiomonas*, *Bacillus*, *Actinobacter*, as well as *Streptomyces* of actinomycetes [26,27,28,29,30]. TTX produced by marine microorganisms is typically a secondary metabolite generated during the stationary phase of bacterial growth. Microbial fermentation experiments have shown that the TTX content produced by bacterial strains is significantly lower than that found in pufferfish [31]. This difference may be attributed to the fact that pufferfish serve as hosts for TTX-producing symbiotic bacteria and can provide the necessary signaling molecules to induce TTX production [32]. In addition, the structure of TTX produced by microbial fermentation was different from that accumulated in pufferfish through food intake [33]. Studies have shown that TTX produced by *Vibrio alginolyticus* was unable to react with monoclonal antibody against TTX in *T. rubripes* [34]. These results suggested that symbiotic microorganisms in pufferfish could produce precursors or analogues of TTX, which need to be transformed and accumulated under the molecular regulation in pufferfish. Even though the molecular mechanism of TTX accumulation and transformation in *T. rubripes* has been reported before [23]. However, our understanding of the TTX-producing bacterial strains and their potential functions remains limited. Meanwhile, 16S rRNA sequencing, which is widely used in microbiota studies, can efficiently identify bacterial species, reflect the diversity and complexity of microbial communities, and compare the differences in microbiota between samples or groups [35]. Conducting a comparative study on the intestinal microbiota of toxic and non-toxic pufferfish using 16S rRNA sequencing could comprehensively identify TTX-related microorganisms.

Our previous study conducted a transcriptomic comparison between toxic and non-toxic *T. rubripes*, identifying functional genes associated with TTX accumulation, translocation, and detoxification [23]. However, the intestinal microbiota contributing to TTX accumulation and their potential functions in pufferfish remain unexplored. In the present study, we characterized the symbiotic intestinal microbiota of toxic (wild) and non-toxic (captive) *T. rubripes* to screen the intestinal microbiota involved in TTX accumulation under different habitats and to preliminarily investigate their potential roles in TTX metabolism in *T. rubripes*.

## 2. Results

### 2.1. TTX Concentration in Wild and Captive T. rubripes

The muscular TTX concentration of wild and captive *T. rubripes* was detected as the phenotypic trait in this study. The TTX concentration in the muscle tissue of wild individuals ranged from 3.9–280 μg/kg, while the TTX concentration in the muscle tissue of captive samples was all below the detectable limit (<1 μg/kg) (Table S1). Therefore, the wild individuals were considered a toxic group, and the captive individuals were considered as non-toxic group in the later comparative analyses. The TTX concentration varies significantly not only among different individuals within the toxic group (80.8 ± 80.5 μg/kg, mean ± SD, *n* = 15), but also between toxic and non-toxic groups under different circumstances. This reflects the TTX content in *T. rubripes* as a complex trait influenced by multiple factors.

### 2.2. OTU Characterization

**Figure 1:**
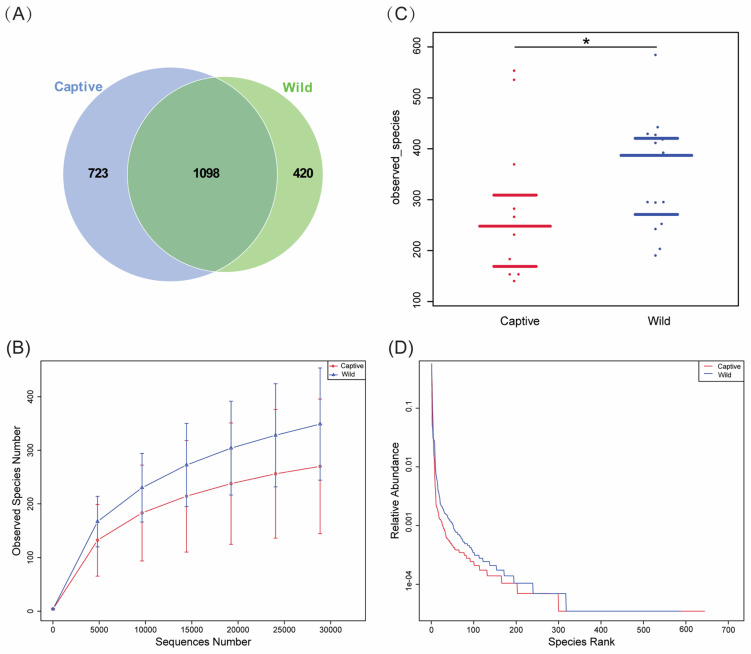
Observed OTUs and species in captive and wild *T. rubripes.* (**A**) Venn diagram of intestinal microbial OTUs in captive and wild groups. Captive represents non-toxic group, while wild represents toxic group. (**B**) Rarefaction curve reflected the observed species number in captive and wild groups. (**C**) The beeswarm plot of observed species differences in captive and wild groups (*p* = 0.038, Wilcoxon test, *n* = 15). The asterisk indicates a statistically significant difference between wild and captive groups. (**D**) Rank abundance curve reflected the relative abundance of captive and wild samples.

From 16S rRNA sequencing, a total of 3,274,513 raw paired-end reads were generated (Table S2). After quality control and removal of chimeric sequences, 1,532,376 effective tags were obtained for further analysis, with an average of 51,079 tags per sample. The average sequence length was 409 bp, and the average effective tags ratio from raw reads was 46.7%. A total of 2241 OTUs were detected using all samples. The captive group detected a higher number of OTUs (1821) in total compared with the wild group (1518). There were 1098 OTUs shared between captive and wild groups, while 723 and 420 unique OTUs were detected in the captive and wild groups, respectively (Figure 1A).

### 2.3. Comparative Assessment of the Microbiota Diversity and Composition Between Captive and Wild T. rubripes

The community abundance and richness of all samples were analyzed by calculating the alpha diversity index. The rarefaction curve showed that the observed species number in each sample of the wild group was consistently higher than the captive group (Figure 1B). The observed species per sample in the wild group was 348 ± 105 (mean ± SD, *n* = 15), and the number in the captive group was 269 ± 126 (mean ± SD, *n* = 15) (Tables S3 and S4). The beeswarm plot reflected that the observed species of the wild group was significantly higher than the captive group (*p* = 0.038, Wilcoxon test, *n* = 15) (Figure 1C). Similarly, the rank abundance curve showed that the community richness in the wild group was also consistently higher than in the captive group (Figure 1D).

**Figure 2:**
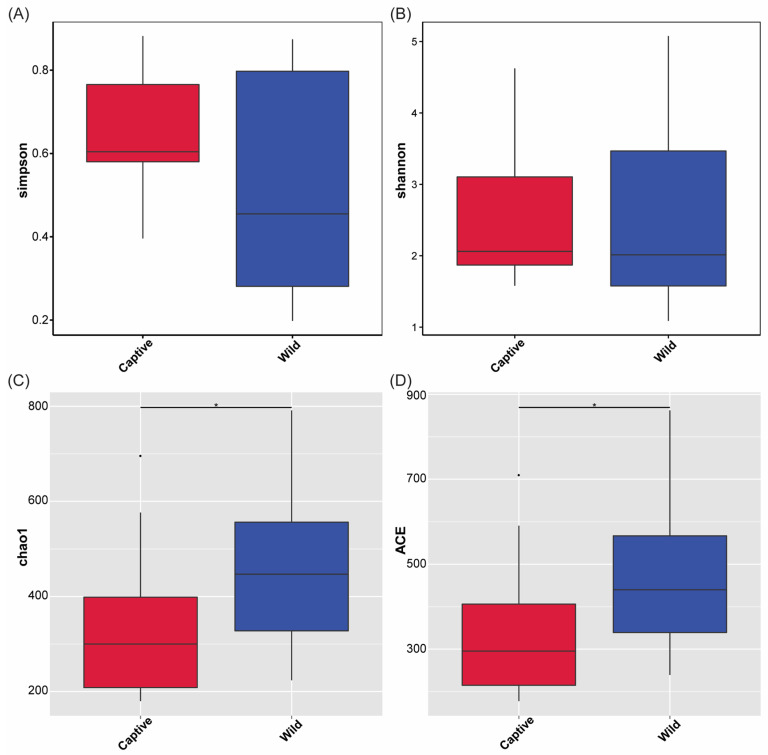
The alpha diversity analysis of intestinal microbiota between wild and captive groups. (**A**) Simpson’s index of diversity (*p* = 0.124, Wilcoxon test). (**B**) Shannon index (*p* = 0.683, Wilcoxon test). (**C**) Chao1 index (*p* = 0.029, Wilcoxon test). The asterisk indicates a statistically significant difference between wild and captive groups. (**D**) ACE index (*p* = 0.033, Wilcoxon test). The asterisk indicates a statistically significant difference between wild and captive groups.

An average good’s coverage of 99.68% indicated most of the intestinal microbiota were identified in this study. After alpha diversity analysis, there was no significant elevation in the Simpson and Shannon indices (*p* > 0.05, Wilcoxon test; Figure 2A,B), which indicated that there were no significant differences in the intestinal microbial community diversity and evenness between captive and wild groups. Whereas, a significant increase in the Chao1 and ACE indices of the community and OTU numbers per sample in the wild group were observed by comparing with the captive group (*p* < 0.05, Wilcoxon test; Figure 2C,D). These results indicated the total number of microbial community and OTUs in the wild group were significantly higher than in the captive group.

### 2.4. Taxonomic Classification of Microbiota

**Figure 3:**
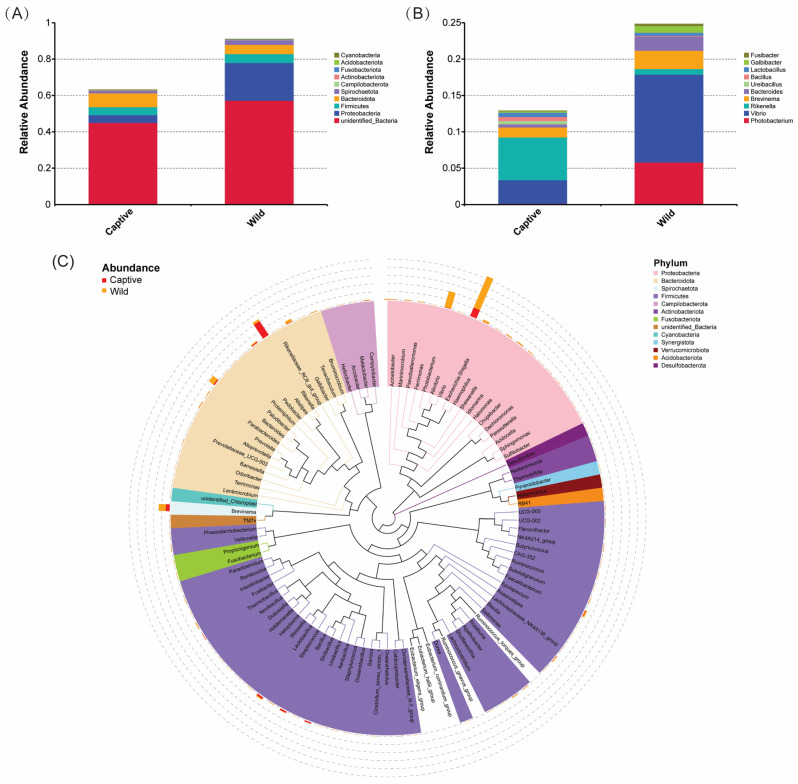
The proportions of intestinal microbiota in the captive and wild group of *T. rubripes*. (**A**,**B**) Relative abundance of top 10 intestinal microbiota at the phylum (**A**) and genus (**B**) level in the captive and wild groups. (**C**) Phylogenetic tree at the genus level in captive and wild group. The colors of the branches and sectors indicated their corresponding phyla, and the stacked bar diagram outside the sectors indicated the abundance distribution of the genus in different groups.

Among all intestinal samples, unidentified bacteria (mainly Campylobacteria at the class level) was the main phylum among the top 10, followed by Proteobacteria, Bacteroidota, Firmicutes, and Spirochaetota. The similar relative abundances were observed between captive and wild groups in the phyla unidentified bacteria (45.02% vs. 57.23%), Bacteroidota (7.60% vs. 5.11%), Firmicutes (4.31% vs. 4.87%), and Spirochaetota (1.39% vs. 2.52%). However, the abundance of Proteobacteria detected in the wild group (20.76%) was about five times that in the captive group (4.34%) (Figure 3A). At the genus level, a total of 563 taxa were identified. In the captive group, *Rikenella* (5.87%), *Vibrio* (3.23%), and *Brevinema* (1.38%) were the top three microbes, which account for more than 1% of total abundance. In contrast, *Vibrio* (12.07%), *Photobacterium* (5.82%), *Brevinema* (2.52%), and *Bacteroides* (1.96%) were the dominant genera in the wild group (Figure 3B). To further study the phylogenetic relationship of microbes at the genus level, the representative sequences of the top 100 genera in both groups were obtained through multi-sequence alignment, and the evolutionary tree was shown in Figure 3C. Among 13 classified phyla, Proteobacteria, Bacteroidota, and Spirochaetota had the highest abundance and the largest difference between groups. Eighteen genera were clustered to Proteobacteria, among which *Vibrio* had the highest abundance, which in the wild group was significantly higher than in the captive group. In the wild group, the second highest abundance genus in Proteobacteria was *Photobacterium*, but its abundance in the captive group was significantly lower. In addition, eighteen genera belonged to the phylum Bacteroidota. In Bacteroidota, *Rikenella* was the most abundant genus in the captive group, and its abundance was significantly higher than that in the wild group. However, *Bacteroides* and *Galbibacter*, as two other dominant taxa, were more abundant in the wild group than in the captive group. Moreover, only one genus, *Brevinema*, was classified into the phylum Spirochaetota; its abundance was relatively high in both groups, although it was somewhat higher in the wild group.

### 2.5. Beta Diversity Analysis

**Figure 4:**
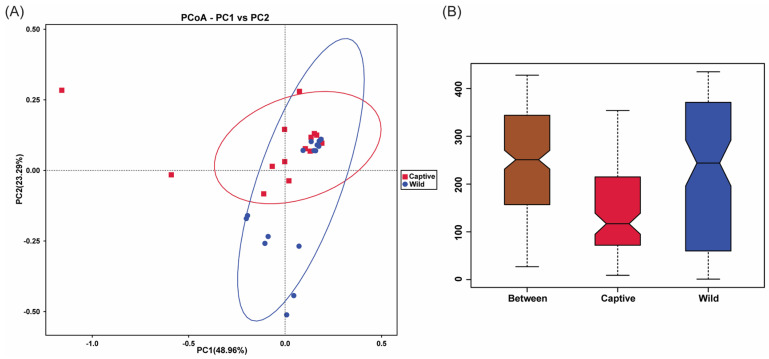
Beta diversity analysis results between captive and wild groups. (**A**) PCoA analysis based on weighted Unifrac distance with cluster. (**B**) The boxplot of Anosim analysis (*r* = 0.262, *p* = 0.001).

According to beta diversity analysis, the composition of intestinal microbiota between two groups was compared and analyzed systematically. The samples of captive and wild groups showed a partly overlapped cluster in the PCoA results (Figure 4A), indicating that there were some differences in microbial community structure between the two groups. Furthermore, Anosim analysis identified that there was an extremely significant difference in the microbial community composition between two groups (*r* = 0.262, *p* = 0.001). In Figure 4B, the notches of the captive group and the wild group did not overlap, indicating significant differences between these two groups.

**Figure 5:**
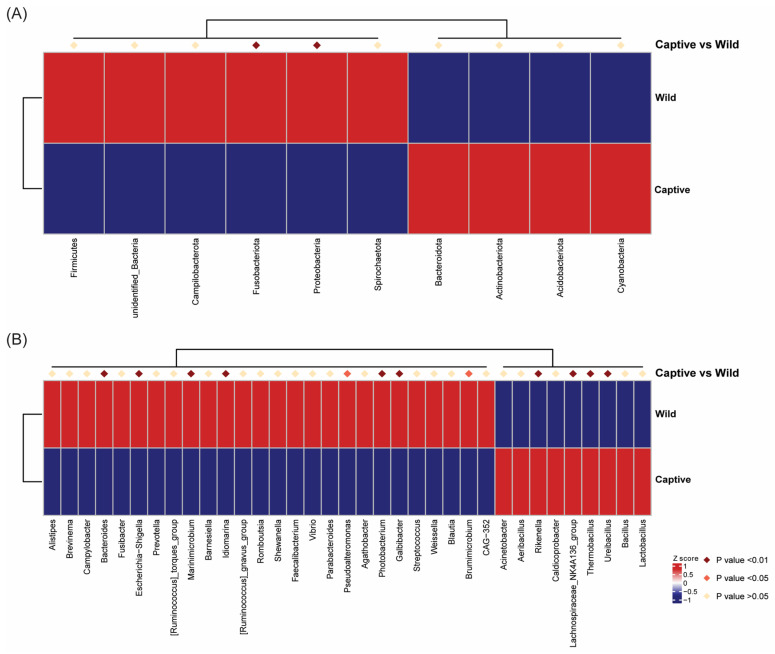
MetaStat analysis results reflected microbes with significant differences between two groups using clustering heatmap at phylum (**A**) and genus level (**B**).

**Figure 6:**
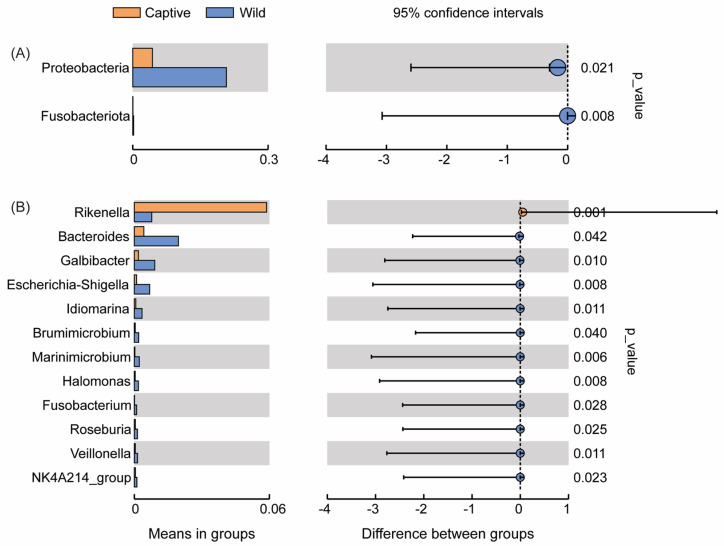
Analysis of intestinal microbiota with significant differences between groups. Barplots with 95% confidence intervals at phylum level (**A**) and genus level (**B**) by *t*-test with *p* < 0.05. The figure on the left represented the microbiota with significantly different abundance between groups, and the figure on the right represented the confidence of differences between groups.

Hypothesis testing was performed on the relative abundance results between groups using Metastats software (http://metastats.cbcb.umd.edu/). Microbiota with significant differences were screened according to *p* values (*p* < 0.05, significant; *p* < 0.01, extremely significant). At the phylum level, Fusobacteriota and Proteobacteria showed extremely different abundance (*p* < 0.01) in captive and wild groups (Figure 5A). At the genus level, ten genera showed extremely different abundance (*p* < 0.01), and two genera showed significantly different abundance (*p* < 0.05) between two groups (Figure 5B). Among these results, *Rikenella*, *lachnospiraceae*_NK4A136_group, *Thermobacillus*, and *Ureibacillus* were extremely significantly more abundant in the captive group, while *Bacteroides*, *Escherichia-Shigella*, *Marinimicrobium*, *Idiomarina*, *Photobacterium*, and *Galbibacter* showed extremely significantly higher abundance in the wild group. T-test results at the phylum level were consistent with Metastats. Compared with the captive group, the relative abundances of Proteobacteria and Fusobacteriota were significantly higher in the wild group (*p* < 0.05; Figure 6A). At the genus level, the top 7 genera (same genera from the Metastats analysis plus *Brumimicrobium)* also exhibited significant abundant differences between the two groups in the *t*-test analysis (*p* < 0.05; Figure 6B).

**Figure 7:**
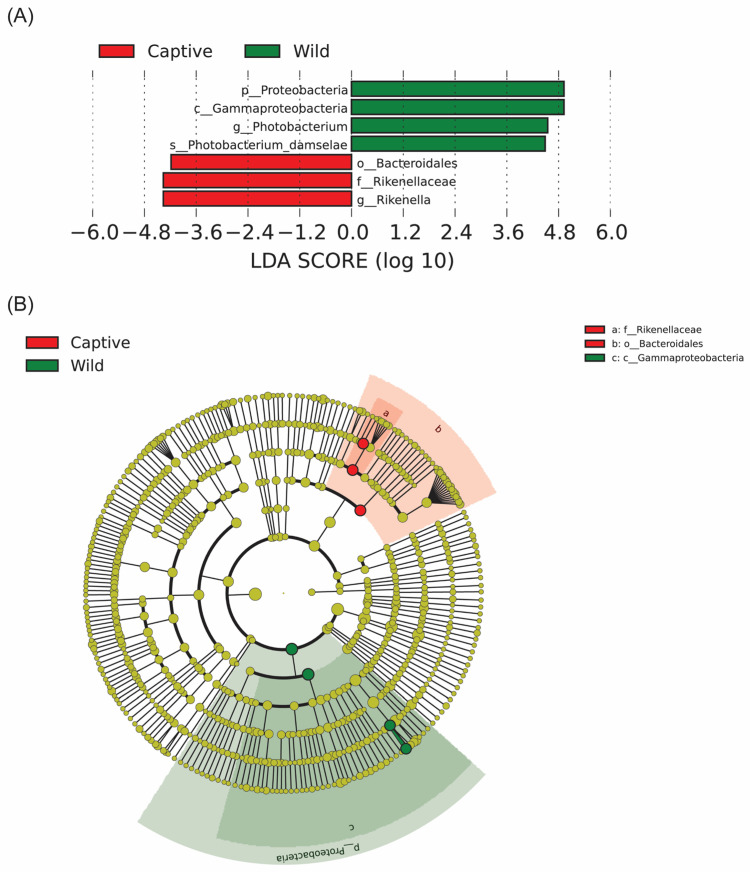
The LEfSe analysis results. (**A**) Histogram of LDA value distribution reflected the Biomarkers with statistical differences in the taxonomic classification (*n* = 15, LDA score > 4). (**B**) The evolutionary cladistic diagram. Circles radiating from the inside out represented taxonomic levels from phylum to genus (or species). Each small circle at a different classification level represented a classification at that level, and the size of the small circle diameter was proportional to the relative abundance size. Color principle: The species with no significant difference were uniformly colored yellow, and the Biomarker of different species was colored with the corresponding group, red: captive group, green: wild group.

LEfSe is an analytical tool for the discovery and interpretation of high-dimensional statistically different biomarkers between groups. From the histogram of LDA value distribution and phylogenetic analysis, we found that Proteobacteria at the phylum level, Gammaproteobacteria at the class level, *Photobacterium* at the genus level, and *Photobacterium_damselae* at the species level were identified as the biomarker in the wild group (*n* = 15, LDA score > 4; Figure 7A,B). Meanwhile, Bacteroidales at the order level, Rikenellaceae at the family level, and *Rikenella* at genus level were screened with an LDA score > 4 in the captive group (*n* = 15).

### 2.6. Function Prediction

**Figure 8:**
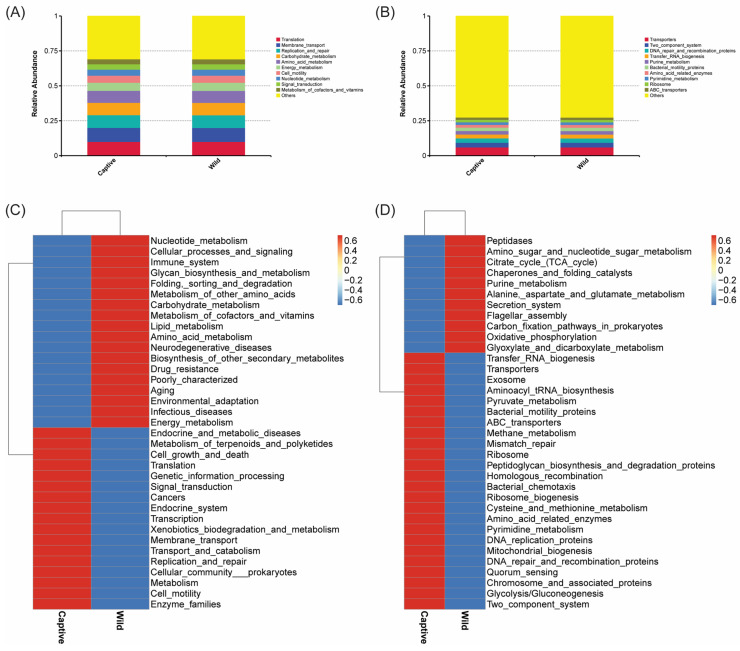
Predicted functions of intestinal microbiota in captive and wild *T. rubripes*. The barplots show the top 10 functional relative abundance at KEGG level 2 (**A**) and level 3 (**B**), respectively. The clustering heatmaps illustrate the KEGG level 2 (**C**) and level 3 (**D**) functional annotations using Tax4Fun.

To predict the functions and their differences in intestinal microbiota in the captive and wild *T. rubripes*, function prediction was conducted by Tax4Fun. The results showed that similar KEGG pathways were observed at level 1, 2, and 3 (Figure S1 and Figure 8A,B), such as “Membrane transport”, “Transporters”, and “ABC transporters”. However, the functional abundances of specific pathways were different between captive and wild populations. For instance, “Biosynthesis of other secondary metabolites”, “Drug resistance”, “Xenobiotics biodegradation and metabolism”, “Membrane transport”, and “Enzyme families” showed significant relative abundance at level 2 between captive and wild groups, while the relative abundance of “Secretion system”, “Transporters”, “Exosome”, and “ABC transporters” was significantly different between captive and wild groups (Figure 8C,D).

## 3. Discussion

TTX is a well-known neurotoxin existing in pufferfish, as well as many other marine organisms [20,36,37,38]. It is distributed in multiple tissues of pufferfish, such as liver, ovary, blood, muscle, etc. [39,40]. Previous studies have shown that the accumulation of TTX in pufferfish was closely related to symbiotic bacteria [26,27]. However, the role of symbiotic bacteria in the production and accumulation of TTX is not yet clear. Therefore, this study compared the gut microbiota of wild toxic and captive non-toxic *T. rubripes* to clarify whether there are characteristic differences in the gut microbiota between toxic and non-toxic pufferfish. In addition, through functional prediction and comprehensive analysis with our previous research results, the indicative bacteria and their possible functions in the production and metabolism of TTX have been explored.

There are abundant and diverse microbial communities in the gut of fish, which is their natural defense system [41,42]. There are complex immune interactions between fish intestinal symbiotic microorganisms and their hosts [43]. The host immune system could recognize and reshape the gut microbiota, while gut microbiota could enhance host tolerance or immune response [44]. Symbiotic microorganisms co-evolve with the host immune system and play a crucial role in the physiological regulation of the host [45]. The diversity and richness of intestinal microbiota are influenced by the genetics, habitat, dietary habits, and health status of the host [46,47,48]. In this study, the intestinal microbiota of toxic and non-toxic *T. rubripes* were compared and analyzed systematically. The results indicated that both the observed bacterial species number and the community richness were higher in the toxic group than in the non-toxic group (Figure 1 and Figure 2), reflecting a more complex intestinal microbiota environment in the toxic *T. rubripes*. The composition of phyla identified in the intestinal contents of toxic and non-toxic groups was almost identical, including Campylobacteria, Proteobacteria, Bacteroidota, Firmicutes, and Spirochaetota (Figure 3A). These results were consistent with recent research on the gut microbiota of juvenile *T. rubripes* [49] and among different pufferfish species [50]. Moreover, similar phyla including Proteobacteria, Firmicutes, Bacteroidota, and Spirochaetota were also found as the dominant bacterial phyla in the TTX-bearing ribbon worms; this may reflect an adaptive strategy of convergent evolution among different species under specific ecological pressures [51]. The common dominant genera identified in both toxic and non-toxic groups were *Vibrio* and *Brevinema* (Figure 3B), which was supported by earlier research related to the gut microbiome’s stability and functionality in the tiger puffer [49,52]. *Brevinema* is a type of spirochete bacteria belonging to the family Brevinemataceae, primarily found in aquatic environments [53]. Based on its spiral shape and motility, it is capable of efficient movement and colonization in complex environments, which may account for its widespread presence in the gut microbiota of pufferfish [54].

Although there were many shared dominant bacteria in the intestinal microbiota between toxic and non-toxic *T. rubripes*, there were also numerous differences between them. For instance, at the phylum level, the abundance of Proteobacteria in the toxic group was approximately five times that of the non-toxic group. Previous research has demonstrated that Proteobacteria was the phylum that contained the most TTX-producing microbes among all [21]. In addition, other reported TTX-producing bacteria primarily belong to the phyla Actinobacteria, Bacteroidetes, and Firmicutes [21]. However, in our findings, differential analysis of the intestinal microbiota between toxic and non-toxic *T. rubripes* identified Proteobacteria and Fusobacteriota as the phyla with significant differences (Figure 5A and Figure 6A). In contrast, Actinobacteria, Bacteroidetes, and Firmicutes showed no significant differences between the two groups, indicating that they may not be the primary phyla involved in TTX accumulation. However, considering the complexity of intestinal microbiota dynamics and functions, it is necessary to further study the specific roles of these microbes. At the genus level, the specific dominant genus in the non-toxic group was *Rikenella*, whereas in the toxic group, the specific dominant genera were *Photobacterium* and *Bacteroides*. Genus *Rikenella* is an important component of the gut microbiota, which plays a significant role in gut health, metabolism, and immune regulation, as well as maintaining intestinal microecological balance through microbial interactions [55]. It is worth noting that, in a previous study, genus *Photobacterium* was found as the predominant genera in the intestinal bacterial community of cultured tiger puffer by contrast [52]. Although we found that the species *Photobacterium damselae* was related to TTX content differences in *T. rubripes*, further investigations are required to verify more bacterial strains and their functions. Under the phylum Proteobacteria, *Vibrio* and *Photobacterium* were the two most abundant bacterial genera in the toxic group, and their abundances were significantly higher than those in the non-toxic group. *Vibrio*, the first isolated TTX-producing bacteria [56], has been found to be associated with TTX production in multiple organisms, such as pufferfish, starfish, xanthid crab, and nemertean worm [30,57,58,59]. In this study, the abundance of *Vibrio* in the wild group was nearly four times higher than that in the captive group, and it was also the most abundant genus in the wild group. However, the difference in abundance did not reach statistical significance in the differential abundance analysis. This could be due to the fact that relative abundance analysis may not fully reflect the true differences, or the differences at the genus level might be diluted by heterogeneity at the species or strain level. In future studies, metagenomic analysis and qPCR could be employed for in-depth investigation and validation of absolute abundance. In the phylum Bacteroidota, genus *Rikenella* showed a significant abundance advantage in the non-toxic group, while *Bacteroides* and *Galbibacter*, also belonging to the Bacteroidota phylum, were significantly more abundant in the toxic group. Genus *Bacteroides*, as well as *Escherichia*, were reported as the allochthonous bacteria in the *T. rubripes* [52]. However, this study is the first to find them to be related to the TTX content difference in *T. rubripes*. Furthermore, this study also discovered for the first time that genera *Marinimicrobium*, *Idomarina*, *Galbibacter*, and *Brumimicrobium* may be responsible for TTX biosynthesis in *T. rubripes*. Additionally, the phylum Fusobacteriota was also identified as a significant differential phylum between the two groups by both Metastats and T-test analyses, with its abundance being markedly higher in the toxic group. Subsequent LEfSe analysis further indicated that the phylum Proteobacteria and the genus *Photobacterium*, as well as the species *Photobacterium damselae,* could serve as the intestinal microbiota biomarker candidates for the identification of toxic *T. rubripes*. Meanwhile, the genus *Rikenella* could be considered as a biomarker candidate to identify non-toxic *T. rubripes*. Moreover, the genus *Pseudoalteromonas*, identified with significant abundant differences between groups and renowned for its ability to produce bioactive compounds and play an important role in marine ecosystems [60], may contribute to TTX bioaccumulation as well. The genus *Pseudoalteromonas* was identified as TTX-producing bacteria in *Nassarius semiplicatus* and *Hubrechtella juliae* and was also found in *Takifugu obscurus* as TTX biosynthesis-related bacteria [30,50,61].

TTX is a bioactive secondary metabolite that can be produced by marine microorganisms [31]. Therefore, based on functional enrichment analysis of the differential gut microbiota identified in this study, we screened level 2 pathways such as “biosynthesis of other secondary metabolites”, “enzyme families”, and “membrane transport” and level 3 pathways such as “secretion system”, “transporters”, and “ABC transporters”, which were closely related to the production and transport of TTX in *T. rubripes*. It should be noted that the pathway “ABC transporters” was also enriched in our previous research, which included three upregulated genes (*abcb6*, *abcb7*, and *abcc5*) in the blood of toxic *T. rubripes* [23]. Likewise, “transmembrane transporter activity” was identified as the biggest pathway nodes, including more than 20 functional related genes in previous research [23]. ABC transporters are a classic membrane protein family responsible for the transport of a wide range of substances across lipid membranes [62]. Studies have reported that in aquatic animals, their function was associated with resistance to xenobiotic toxic substances [63,64]. Combined with our research findings, ABC transporters may play significant roles in the production and transport of TTX in both symbiotic microorganisms and hosts. A possible hypothesis is that TTX produced by the gut microbiota enters the host’s body and reaches different tissues through the circulatory system, thereby regulating the expression of host genes [65]. In turn, the expression of host genes may alter the intestinal environment, thus affecting the growth of specific microbial communities. Additionally, dietary factors may also influence the expression of host genes and the composition of gut microbiota. Therefore, the exact correlation and co-regulatory mechanisms between host and gut microbiota in TTX production and accumulation remain to be investigated in future studies.

## 4. Materials and Methods

### 4.1. Sample Collection and TTX Detection

All animal procedures involved in this study were approved by the Animal Care and Use Committee of the Centre for Applied Aquatic Genomics at the Chinese Academy of Fishery Sciences before sampling. All procedures and animal handling were conducted in accordance with approved guidelines, with every effort made to minimize potential suffering.

Wild (toxic; body length, 16.8 ± 1.2 cm; body weight, 111.2 ± 29.0 g) and captive (non-toxic; body length, 17.1 ± 1.3 cm; body weight, 130.5 ± 22.0 g) *T. rubripes* were collected from the Bohai Bay (Tangshan, Hebei, China), then temporarily reared in the marine fish breeding laboratory of Beidaihe Central Experimental Station, Chinese Academy of Fishery Sciences. Captive *T. rubripes* were fed a combination of TTX-free natural food sources (including rotifers, brine shrimp, and fresh fish), along with a TTX-free commercial feed provided by Qingdao Saigelin Aquatic Products Technology Co., Ltd. (Qingdao, China) from the time of hatching. Wild and captive fishes were acclimated separately in the same-sized seawater tanks at 20 °C without feeding for three days. Then, fifteen individuals from each tank (approximately 30–40 fish per tank) were randomly collected for sampling. For TTX detection and DNA extraction, the intestinal contents and muscle tissues were collected from each individual. The samples used for TTX detection were taken from the dorsal muscle, with each sample weighing no less than 15 g. The samples were flash-frozen in liquid nitrogen, then stored at −80 °C until further use. The TTX detection was conducted by Meizheng Bio-Tech Co., Ltd. (Beijing, China), as our previous study described [23]. Briefly, the TTX concentration of muscle tissues was detected by the liquid chromatography-tandem mass spectrometry (LC-MS/MS) method, according to the China National Food Safety Standards GB 5009.206-2016: Determination of tetrodotoxin in aquatic products [66].

### 4.2. DNA Extraction, Amplification and Sequencing

Microbial DNA from the intestinal content of toxic and non-toxic *T. rubripes* was extracted using the QIAamp PowerFecal Pro DNA Kit (Qiagen, Hilden, Germany) in accordance with the manufacturer’s protocols. The primers used for PCR amplification of the 16S rRNA V3–V4 region were 341F: CCTACGGGNGGCWGCAG and 806R: GGACTACHVGGGTATCTAAT [67]. The PCR amplification conditions were as follows: 95 °C for 3 min, followed by 30 cycles of 95 °C for 30 s, 50 °C for 30 s, 72 °C for 1 min, and finally 72 °C for 7 min. Each 20 μL reaction contained 10 μL premixed Taq (Takara, San Jose, CA, USA), 1 μL of forward and reverse primers each (10 μM), 2 μL gDNA, and 6 μL ddH_2_O. The amplicons were purified by AMPure XP Beads (Axygen, Union City, CA, USA) and quantified by Qubit 2.0 Fluorometer (Invitrogen, Carlsbad, CA, USA). The purified amplification products were sent to the Novogene company (Tianjin, China) for 16s rRNA sequencing using the Illumina NovaSeq platform PE250 strategy.

### 4.3. Bioinformatics and Statistical Analysis

The raw data of each sample were separated according to barcode sequence and PCR amplification primer sequences. Paired-end clean reads were merged to raw tags using FLASH (version 1.2.7) [68]. The filtering, dereplication, denoising, and chimera sequence removing were conducted by using QIIME (version 1.9.1) [69] and Vsearch (version 1.9.6) to generate effective tags [70]. Effective tags were clustered into operational taxonomic units (OTUs) with 97% identity by Uparse software (version 7.0.1001) [71]. The sequence with the highest frequency in a cluster was selected as the representative sequence to perform species annotation. The annotation analysis was conducted using the Mothur method and SILVA138 database [72]. Taxonomic analysis was performed using MUSCLE (version 3.8.31) with a naive Bayesian classifier [73].

Alpha diversity indices (including Chao1, ACE, Shannon, and Simpson) were calculated using QIIME (version 1.9.1) [69]. QIIME was also used to calculate weighted Unifrac distance and construct a UPGMA cluster tree. The rarefaction curve and rank abundance curve were drawn using R software (version 2.15.3). PCoA (principal co-ordinate analysis) analysis based on weighted Unifrac distance was processed using R software WGCNA, stats, and ggplot2 packages. The differences analysis between toxic and non-toxic groups of alpha diversity and beta diversity indices was assessed using the Wilcoxon test in R software (version 2.15.3). The Anosim analysis, reflecting the significance of the difference between groups, was conducted using the R vegan package. To further excavate the microorganisms related to the TTX content difference between groups, T-test, MetaStat analysis, and LEfSe (LDA effect size) analysis were performed using R (version 2.15.3), Metastats (http://metastats.cbcb.umd.edu/), and LEfSe software (version 1.0) [74,75].

### 4.4. Functional Analysis

Tax4Fun [76] was used to predict the function of intestinal microbiota in different groups by the nearest neighbor method based on a minimum 16S rRNA sequence similarity. The specific method consisted of extracting the whole genome 16S rRNA gene sequence of prokaryotes from the KEGG database and comparing it to the SILVA SSU Ref NR database (BLAST bitscore > 1500) using the BLASTN algorithm to establish a correlation matrix. The whole genome functional information of prokaryotes in the KEGG database annotated by UProC and PAUDA corresponds to the SILVA database to achieve functional annotation of the SILVA database. The functional relative abundance distribution and clustering analysis were proceeded using R software.

## 5. Conclusions

In this study, we characterized the symbiotic intestinal microbiota of toxic (wild) and non-toxic (captive) *T. rubripes*. Potential intestinal microbial communities involved in the production and accumulation of TTX were identified by comparing two groups of *T. rubripes* with different TTX contents. We also preliminarily explored their potential functions in TTX accumulation and metabolism within *T. rubripes*. This study revealed that bacterial genera potentially associated with TTX production and accumulation mainly belong to the phyla Proteobacteria and Bacteroidota. The genera *Vibrio* and *Photobacterium* from Proteobacteria and the genus *Bacteroides* from Bacteroidota were significantly more abundant in the wild population than in the captive group. Combined with existing findings, we hypothesize that these genera play important biological roles in TTX accumulation within pufferfish. Meanwhile, although the genera *Marinimicrobium* and *Idomarina* from Proteobacteria and the genera *Galbibacter* and *Brumimicrobium* from Bacteroidota had relatively low abundance, they exhibited significant differences between the two groups, suggesting that these genera may also be involved in TTX accumulation. Additionally, enrichment analysis of differentially abundant microbial communities identified pathways closely related to secondary metabolite biosynthesis, secretion, and transport, implying that the potential functions of these differentially abundant communities are associated with TTX biosynthesis, secretion, and transport. However, the specific species/strains and their functions still need to be confirmed through metagenomic, metabolomic, and molecular cellular functional experiments.

## Data Availability

The 16S rRNA sequencing data reported in this research are available at the Sequence Read Archive (SRA) under accession number PRJNA1223122.

## Supplementary Materials

The following supporting information can be downloaded at: https://www.mdpi.com/article/10.3390/md23040140/s1, Figure S1: The barplot of KEGG 1-level top10 functional relative abundance. Table S1: Muscular TTX concentration of wild and captive *Takifugu rubripes*. Table S2: Overall results of 16S rRNA sequencing Table S3: Summary of the Alpha diversity index Table S4: Summary of the Alpha diversity index in group.
