# Supplementary figures and images for: Comparative Analysis of Intestinal Microbiota Between Tetrodotoxin-Containing and Tetrodotoxin-Free Takifugu rubripes
Source: Mar Drugs. 2025 Mar 24;23(4):140. doi: 10.3390/md23040140 (PMC12028943; doi:10.3390/md23040140)

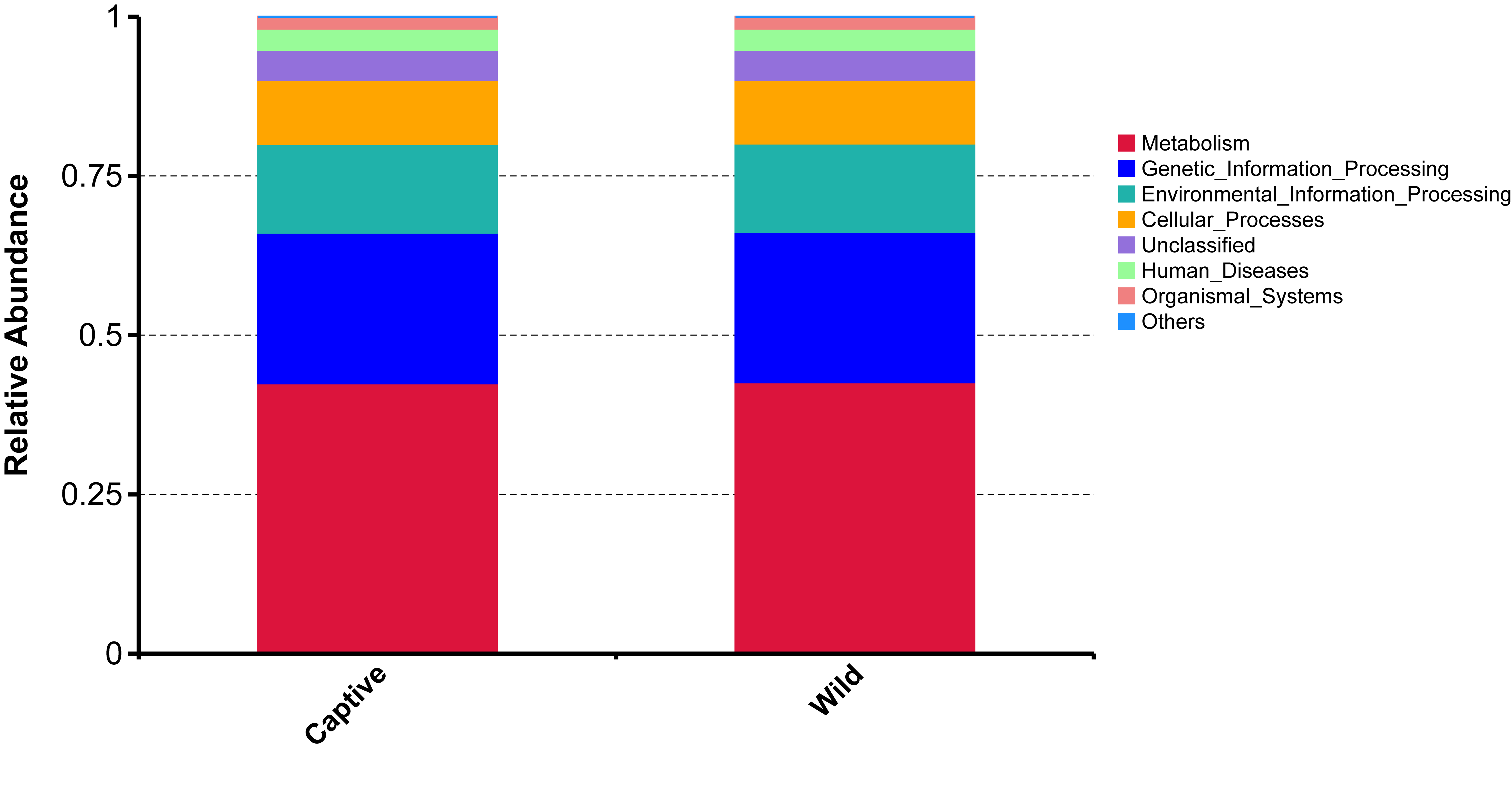

Supplement: Supplementary file 1 [file marinedrugs-23-00140-s001.zip › marinedrugs-3491259-supplementary/Fig S1.tif]
